# Livestock hauler and dairy farmer perspectives about cull dairy cattle transport and cattle transport regulations in British Columbia, Canada

**DOI:** 10.1017/awf.2023.36

**Published:** 2023-06-01

**Authors:** Christine Kuo, Marina AG von Keyserlingk

**Affiliations:** Animal Welfare Program, Faculty of Land and Food Systems, The University of British Columbia, 2357 Main Mall Vancouver, BC, Canada, V6T 1Z6

**Keywords:** Animal welfare, attitudes, cull cows, drover, hauler, regulations

## Abstract

Dairy cows are usually culled and transported from the farm when they no longer meet the farm’s standards for production or are not needed for milk production. Some cows are transported while in poor condition and may deteriorate further during transport. In February 2020, Canadian federal animal transport regulations were revised with the aim to minimise risks to livestock during transport; changes that may impact cull dairy cows included defining compromised cattle and limiting their maximum transport time. This study conducted semi-structured interviews with dairy farmers (n = 6) and cattle haulers (n = 4) in British Columbia, Canada, to gain an in-depth understanding of the effect of the regulations on their practices when shipping and transporting dairy cows to slaughter. Interviews were transcribed in Otter.ai and thematically coded in NVivo 12. While farmer and hauler participants recognised the importance of animal welfare during transport and described practices such as shipping mobile animals to reduce the risk that cows would become non-ambulatory during transport, they also described little change in shipping and transport practices due to the new regulations. Among interviewed participants, barriers to compliance with the regulations appear to be low knowledge of, and mixed or negative attitudes towards the regulations. Participants also described how they felt a lack of communication along the transport chain and limited transport and slaughter infrastructure made compliance difficult. Possible suggestions to remedy these barriers include providing educational resources about the regulations and encouraging communication about cow fitness for transport between responsible parties in the transport chain.

## Introduction

Cull dairy cows are dairy cows that are removed from the herd. In 2021, 32% of dairy cows, or 246,331 cows, were culled in Canada (Canadian Dairy Information Centre [CDIC] 2021). Cull cows are typically slaughtered and may be transported through one or multiple assembly points such as livestock yards, auction markets, or buying stations before arrival at the slaughterhouse (Stojkov *et al.* 2018).

In Canada, dairy cows may be culled due to issues such as reproductive health, poor fertility, sickness, injury, personality, and milk quality (CDIC 2021), or due to fluctuations in the milk quota (Marshall *et al*. 2022). The former reasons may impact the welfare of cull dairy cows during transport and increase their vulnerability to transport stressors (for a review, see Cockram 2021). For example, Stojkov *et al.* (2020) observed cull dairy cows from 20 farms in British Columbia and reported that 16–26% of these cows were shipped with health conditions that can impact fitness for transport, including thinness, lameness, and poor udder condition. Cull dairy cows are also at risk of deteriorating further during transport, such as becoming clinically or severely lame, experiencing milk leakage, or experiencing wounds during transport (Dahl-Pedersen *et al.* 2018b). Collectively, the available evidence highlights the animal welfare challenges impacting cull dairy cows and how these challenges can be exacerbated due to transport.

However, as Edwards-Callaway *et al.* (2019) summarised about cull dairy cow transport in the United States, there are currently no financial penalties for shipping and purchasing unfit dairy cows, and these cows continue to be sold and marketed. Reasons provided by these authors for the continued marketing of vulnerable dairy cows include: farmers wishing to avoid the cost of euthanasia or attempting to recoup some of the cow’s value through sale; haulers choosing to transport compromised animals in order to maintain business relationships with farmers; and purchasers taking advantage of the potential profits that are available for slaughtered cull dairy cow carcases. Unfit cattle may also continue to be sent to slaughter for reasons such as: dairy farmers may be unaware of the welfare issues associated with transport; a lack of enforcement of laws that protect animal welfare during transport; inadequate evaluations of fitness for transport and euthanasia-decision making; and failure to perform proactive culling (Cockram 2021). Despite these challenges, there is a need for ensuring that animals are transported while in good health so that they may be able to cope with transport-related stressors and remain in good condition throughout the journey, thereby protecting their welfare.

Transport of vulnerable dairy cattle is particularly difficult in British Columbia because of limited provincial slaughter capacity and infrastructure for cull dairy cows, meaning that direct shipment to slaughter is often not possible and most dairy cows are therefore transported long distances for slaughter (Stojkov *et al.* 2018, 2020). One study followed the journey of cull cows from 20 dairy farms in the lower Fraser Valley region of British Columbia and found that 80% were slaughtered in the United States, 11% in Alberta, and only 9% were slaughtered within British Columbia, and these animals spent an average of 82 h in the transport and marketing system (Stojkov *et al.* 2020). During this time, cows may be subject to stressors including shipping delays, mixing with animals from different farms, and frequent handling due to reselling and movement through the auction market system (Stojkov *et al.* 2018).

Animal transport is an area of public concern in Canada (Spooner *et al.* 2014) and in other jurisdictions, as evidenced by the formation of the Committee of Inquiry on the Protection of Animals during Transport by the European Parliament in 2020 in response to concerns about animal transport (Bachelard 2022). In Canada, livestock transportation falls under federal jurisdiction. Animal transport laws were revised and updated in 2020 with a two-year grace period before enforcement by the Canadian Food Inspection Agency (CFIA). Changes in these laws with respect to cull cows included that compromised cattle are now defined as having certain health conditions including lameness or being in heavy lactation (Health of Animals Regulations [HAR] Part XII 136[1] 2022). They must be isolated from other animals and transported to the nearest location for slaughter or care (HAR Part XII 140[1] 2022). Compromised cattle cannot be transported for longer than 12 h without feed, water, or rest, and a rest stop must be at least 8 h long (HAR Part XII 152.2 [1] 2022). Additionally, lactating animals must be milked at appropriate intervals to prevent mammary engorgement (HAR Part XII 142; 152.2 [1] 2022), and the regulations include specific language limiting the use of electric prods (HAR Part XII 144[1] 2022). Prior to 2020, there were no special provisions for compromised cows. All cattle that were not considered unfit under the regulations could be transported for up to 52 h without feed, water, or rest, provided they reached their final destination within 52 h; if they would not reach their final destination in 52 h, transport was limited to 48 h without feed, water, or rest (Government of Canada 2016).

Given that little research has focused on the attitudes of dairy farmers and cattle haulers towards cull dairy cow transport, this research aimed to gain an in-depth understanding of the attitudes of farmers and haulers, and their decision-making process as to whether a cow is deemed fit for transport. As the new Canadian federal regulations came into effect in 2022, we also aimed to gain a preliminary understanding of the impact of the updated Canadian federal transport regulations on dairy farmer and cattle haulers’ knowledge of cull dairy cow transport, as well as their practices when shipping and transporting cull dairy cows. Although there is existing research on the transport of cull dairy cattle in British Columbia (Stojkov *et al.* 2018, 2019, 2020) and the impact of the regulations on cull dairy cow transport in other provinces in Canada (Hendricks *et al.* 2023), this is the first study to interview dairy farmers and haulers in British Columbia in the context of the new regulations.

## Materials and methods

This study was approved by The University of British Columbia Behavioural Research Ethics Board (protocol #H22-01887). All participants provided verbal consent and were offered a $C20 digital gift card as thanks for participation, although some participants declined the gift card.

Semi-structured qualitative interviewing was used to ascertain British Columbian cattle hauler and dairy farmer perspectives on the regulations. Qualitative interviewing was selected because it can assess stakeholders’ beliefs, motivations, and values (Berkwits & Inui 1998). Semi-structured interviewing was chosen so that the structure of the interview may be flexible and participant-led, allowing participants to freely describe their own perspectives and experiences (Lewis-Beck *et al.* 2011).

The question guide for haulers was developed first and based, in part, on the question guide by Hendricks *et al.* (2023). The question guide by Hendricks *et al.* (2023) focuses on surplus calves; however, because the current study deals with cull cows, questions specific to cull cows were identified from discussions between the researchers, literature reviews, and close reading of the regulations. The final hauler question guide covered hauler training, the dairy cow transport process, and the regulations (see Table 1 in Supplemental materials; https://doi.org/10.5683/SP3/E3MIHO). The question guide for farmers was developed after all hauler interviews had taken place and was adapted from the hauler guide. The final farmer question guide covered decision-making when culling cows, the relationship with one’s cattle hauler, knowledge of the cull cow transport chain, and the regulations (see Table 2 in Supplemental materials; https://doi.org/10.5683/SP3/E3MIHO). The questions regarding the regulations were similar between the hauler and farmer question guide and discussed knowledge of the regulations, the impact of the regulations on their work, and attitudes towards the regulations. The other questions varied between the guides because they were specific to work as a farmer or hauler.

Interviews took place between August and November 2022. Inclusion criteria for haulers required them to be based in British Columbia and have transported dairy cattle at least once commercially. A convenience sample of four livestock haulers were interviewed: two were sourced from known contacts of the researchers and one was recommended by snowball sampling from a participating dairy farmer. Finally, eleven haulers were contacted from two publicly facing websites and one agreed to participate. Inclusion criteria for farmers required that they were dairy farmers in British Columbia. A total of six farmers were interviewed; four were sourced from known contacts within our research group and four were recommended through snowball sampling from participating dairy farmers.

Interviews lasted from 24 to 61 min (average of 40 min). One interview was conducted over Zoom (Version 5.11.10, 2022), and the remaining nine were conducted via telephone according to the participants’ preference. Audio recordings of the interviews were transcribed using a professional online transcription service (Otter.ai). These transcriptions were checked for errors and de-identified. Prior to data analysis, each participant was emailed a copy of the transcript and could request modifications. With the exception of one participant requesting that a portion of the interview be ‘off the record’ during the interview, no participant requested any modifications. Each participant was assigned a unique identifier that denoted whether they were a hauler (T) or farmer (F) and three randomly generated numbers (e.g. T123). Although some hauler participants also had their own farm, these participants are identified only as haulers and were interviewed using the hauler interview guide. Some farmers hauled their own cattle to the stockyard; these were only identified as farmers and interviewed using the farmer interview guide as they did not haul cattle for other dairy farmers.

Transcripts were thematically analysed following Braun and Clarke (2006) using NVivo software (Version 12 2022). Thematic analysis is a flexible research tool to identify, analyse and report patterns in data (Braun & Clarke 2006). Each transcript was read to identify patterns, inductively develop initial descriptive codes based on these patterns, and assign these codes to relevant text segments. Only segments of the interviews that were related to the research questions were coded, as the coding process was intended to condense the data (Elliott 2018). These codes were then reviewed, refined, clustered, and sorted into themes. Themes were reviewed, then organised and defined in a codebook. Interviews were then re-coded according to the final codebook. Separate codebooks were made for the hauler and farmer interviews. The hauler and farmer codebooks had the same primary themes, but different subthemes. For reporting results, quotes were condensed by removing verbal ticks and repetitive words. Square brackets (i.e. […]) indicate where quotes have been modified.

## Results and Discussion

Three themes emerged during interviews with haulers and farmers: knowledge and attitudes towards the regulations; cull cow management and responsibility; and the cull dairy cow transport chain in British Columbia.

### Knowledge and attitudes towards the regulations

All participants were aware of the change in regulations, though in varying levels of detail. Participants also described a range of attitudes towards the regulations, with some praising them for protecting haulers and animal welfare, while others voiced frustrations with government regulation and questioned whether the regulations actually improved animal welfare.

All farmer participants described learning about the regulations from the British Columbia Dairy Association (BCDA), a producer group with mandatory membership that represents all dairy farmers in the province. Meanwhile, hauler participants described learning about the regulations from sources such as industry boards, livestock inspectors, farmers, or the British Columbia Livestock Association Cooperative which runs livestock yards in the province. As an official producer group in British Columbia, it is unsurprising that the BCDA was influential in informing farmers of the changes in the transport regulations. In contrast, haulers do not have an official provincial nor federal association equivalent to the BCDA and thus were likely limited to indirect sources of information such as farmers or other industry sources. The lack of affiliation of the hauling industry with an organisation may have increased the challenges associated with educating haulers on transportation regulations. Additionally, the variation in information sources for haulers may increase the risk of confusion about the regulations because there may be inconsistencies between the sources. Research on healthcare professionals by Matthys *et al.* (2019) suggests that members of professional organisations may feel that active participation in a professional organisation can aid them in staying up-to-date with an industry and profession because the organisation may offer resources like educational services and information on legislation. One possible interpretation from this is that the creation of such an organisation for haulers may aid in education efforts.

All participants generally had little detailed knowledge of the regulations. In the case of the farmers, some were aware of a 12-h limit, though mostly in the context of calf transport and not in regards to compromised adult cattle. For example, farmer F398 briefly described the new maximum transport time for cows but knew more about the impact of the regulations on calf transport:

“*I can’t say a whole bunch offhand that comes to mind. Because I have read them. There’s, you know, limits on how far they can travel without rest stops. And cows need to be a certain age before they can move any distance.* [They need to be] *nine days* [old] *or whatever to go through a gathering place and older if you want to move them like 12 hours or whatever. Beyond that, as far as specifics, I can’t say offhand, no.*”

Farmers may be more familiar with the calf component of the new regulations because they were simply understood as limiting the transportation of calves of a certain age and thus were easy to convey during the interviews. In contrast, the definition of a compromised cow in the new regulations includes mention of nine specific health conditions (i.e. lameness, bloat) whilst an unfit cow now includes 22 specific health conditions (i.e. non-ambulatory, dehydrated) (Government of Canada 2016), making the description of compromised and unfit cows more complicated to recall. Little knowledge of the regulations does not seem to be unique to British Columbia. An interview-based study in Atlantic Canada provided additional evidence that some haulers are unclear on the details of what is allowed when transporting dairy cattle (Hendrick *et al.* 2023).

Although all four haulers knew about the existence of the new regulations, they were rarely able to provide details: *“I know I have heard that there’s something out there but no, other than that I don’t really know what’s exactly in it, no”* (T249). One hauler (T023) knew of the existence of the regulations but asked the interviewer for details to share with a farmer client who was curious about the maximum transport time:

“[…] *In the past* […] *there’s no regs that says you have to have them off in a 24 hour, 48 hour, or whatever, period. So I don’t know.* […] *Have you heard at all what they’re doing or have done about how long animals are supposed to be on cattle liners?”*

One hauler (T177) did mention provisions for compromised animals in the regulations but did not define the conditions:

“*Yeah, so there’s two, I’m trying to think of the term of the other… You’ve got compromised and you’ve got, basically, can’t remember the other term, but* […] *under those circumstances, as I remember it,* […] *they would be in your stock trailer, but they would be separate, you know, in order to ship them, some have to go direct to slaughter and you know, some can’t go through an auction yard.”*

After introducing the new regulations in February 2020, the CFIA allowed for a two-year transition period before enforcement to allow for time to educate haulers, farmers, and other related professionals about the new regulations (Government of Canada 2022). The results from our study support the work of Marshall *et al.* (2022) and Hendricks *et al.* (2023) in suggesting that these education efforts do not seem to have been successful. Although all participants knew of the existence of the regulations, they typically did not know the details. It is possible that the provision of learning resources about the regulations, particularly about compromised and unfit cattle, may help farmers and haulers become more knowledgeable about the regulations and protect the welfare of cull cows.

Participants also expressed mixed attitudes towards the regulations. Some haulers expressed positive attitudes towards the regulations, such as hauler T023:

*“I know they’ve changed a lot of rules and regulations on that which are great. For a while, everything went to the auction barn, didn’t matter who was half dead or not or alive. And we just had to put a stop to that because they die in our liner, and then it’s our insurance to pay for it. And I just think that’s wrong, right? So as a driver, you just say, hey, if that’s not a healthy cow, I’m not taking her.*”

This positive sentiment was echoed by farmer F663 who stated that:

“*I trust in the science and if they tell us that animals are compromised, are acutely uncomfortable after spending that many hours in the truck, I mean, just take a look at yourself. Can you stand in one spot? You know at some point you say I do need a break and why should an animal be any different?* [If] *this is what the science tells us, I’m not going to raise a lot of* [fuss].”

However, not all were in agreement that the changes were positive. Hauler (T249) expressed frustrations towards the regulations:

*“…We do what we do. We know how to have common sense. We know how to haul cattle, and we don’t value people trying to tell us how to haul cattle.* […] *And you know what,* […] *I really do get it.* […] *You get those one-off stories of abuse. It’s something so now all of a sudden, now a whole industry has to change. We have to have new regulations and new rules and new everything so that we’ll never have something like that happen again. I struggle with that theory.*”

Participant (T249) felt transport issues should be dealt with on a case-by-case basis because they felt that changing the entire regulatory system created more work for haulers:

“*OK, if somebody is caught that’s hauling and they found abuse or they’re taking animals in a inhumane way, deal with that hauler. Whatever, fine them I guess or whatever. But don’t go somehow change all the rules and hope that you can somehow change every single hauler to do* [that]. *They were likely doing the right thing already but* [changing regulations] *make*[s] *it more difficult* [for them] *to do their job and do it properly.*”

Disregard for the regulations by some haulers was also raised by Hendricks *et al.* (2023) where some haulers working in Atlantic Canada openly stated that they would simply defy the regulations by working around the CFIA. In the current study, one farmer (F398) also expressed concerns that the regulations may harm animal welfare because animals will need to be reloaded more often, which may lead to poor animal handling practices:

“*I think a lot of* [the regulations] *are probably a little bit overdone. For the rest stops, for instance, they’re not always good. I mean, they can be stressful too, because you’re unloading and reloading and generally once they’ve been on a truck for a while, they’re not that keen on going back on, so then there’s probably some stuff happening that maybe shouldn’t be happening when they’re trying to reload. So, I think within reason,* […] *anything under, say, 16, 18 hours, it’s probably less stressful to just stay* […] *on the truck and get there and be done with it rather than adding in extra stress with a stop.*”

Concern that mandatory rest stops could harm animal welfare was also raised by haulers interviewed by Hendricks *et al.* (2023). Research on the efficacy of rest stops is mixed. While unloading may allow animals to access feed and water and recover from the journey if the stop is long enough, it does come with additional stress associated with loading, unloading, and potentially being mixed with unfamiliar animals in unfamiliar environments (Cockram *et al.* 2000). Whilst some have argued that rest stops extend an already long transport duration (Cockram & Mitchell 1999; Cockram 2007), failure to do so extends the length of time an animal is prevented from eating, drinking, and resting. In a recent study, Meléndez *et al.* (2020) concluded that rest stops have little effect on behavioural and physiological indicators of calf welfare when 7–8 month old conditioned beef calves, which were weaned, castrated, dehorned, ear-tagged, adapted to eat grain from a feed bunk, and adapted to drink from a water trough 18–26 days prior to transport, were transported 12 or 36 h, rested for 0, 4, 8 or 12 h, and then transported for another 4 h. However, these results should be viewed with caution given that the authors only examined lying and feeding behaviour in the days following transport and failed to evaluate behaviour during the resting period, loading, and unloading events. Additional research is needed on the effect of transport and rest stops on cattle in general, and specifically on cull dairy cows and dairy calves, as well as the effect of rest stops during journeys with multiple stops.

One of the farmers (F398) hypothesised that the new regulations were developed by animal rights activists to harm animal agriculture:

“*I’m afraid it’s probably some animal rightist end of things. That they figure it’s a good idea* […] *to make things a little more onerous to hope that* [it will] *cut back on the animal agriculture thing. And I’m not sure how much real science or animal knowledge went into that. I understand that* […] *dairy industry people are involved too, but* […] *I think a lot of it is just not really thinking through all the consequences. You know, it sounds good, give them a break after 12 hours or whatever. But if you think a little bit further* [about] *the stresses of reloading and stuff, then it doesn’t sound like quite as good an idea anymore.*”

One farmer also suggested that the regulations were not relevant to British Columbia because they believed cows were not transported for more than 12 h: “*It’s just that I don’t think cows are being shipped for 12 hours to get to a slaughter plant or going to Seattle or wherever*” (F833). Similarly, two of the four hauler participants stated that the duration that they transported cattle was always less than 12 h. Hauler T543 described how they felt their hauls were unaffected by the regulations because they did not transport cattle for long distances:

*“*[…] *Hauling a certain amount of hours and all that? And making sure that you rest the animals and all that?* […] *That doesn’t really come into effect for me because* […] *they’re not on the trailer that long with me.* […] *It would be a problem for me if I was doing like, say here to Alberta or something myself. But to me, it doesn’t. Those rules don’t really affect me.”*

Though these participants focused on the 12-h time limit for compromised cows, the regulations include other provisions such as that compromised cows must not be taken to an assembly point. Thus, even if compromised cattle are reported to be transported for under 12 h, the regulations still impact the transport of compromised cows and are relevant to British Columbia. Additionally, the journey of a cull dairy cow is typically composed of multiple stops and transfers in ownership, and cull dairy cows in British Columbia may be in the marketing system for an average of over three days with many being transported out of the province (Stojkov *et al.* 2020). Informing producers and haulers about typical transport times and distances for cull cows, including that the full journey may go beyond the borders of British Columbia, may help them see the relevance of the regulations.

Skogstad (2003) argued that the legitimacy of a law is made of input factors such as how the law was developed, and output factors such as the effectiveness of the law. Legitimacy can also be described as a social process whereby new laws must gain social acceptance and be seen as commonly accepted, valid practices, even if individuals may disagree with them (Johnson *et al.* 2006). In this study, some participants questioned the effectiveness and necessity of the regulations, potentially undermining their legitimacy, which in turn may weaken compliance. Braun and Busuioc (2020) suggest that careful engagement with stakeholders around the development and implementation of regulations can aid in bolstering regulatory legitimacy. Such engagement with cull dairy cow transport stakeholders, including dairy farmers and cattle haulers, could potentially improve perceptions of and compliance with the regulations.

### Cull cow management and responsibility

The six participating farmers were asked to describe the process of deciding if and when to cull and ship a cow, while the four participating haulers were asked to describe the transport process from pick-up at the farm to delivery. Farmers discussed fitness for transport and management practices before shipping cows. Haulers also discussed fitness for transport and additional practices that they use to support animal welfare (i.e. ensuring trailer cleanliness) during transport.

Farmers typically defined fitness for transport as a mobile, healthy cow. One farmer stated that this meant “*She has to be able to walk and get on the truck, not have a fever and be a healthy cow*” (F663). Another farmer highlighted the importance of mobility: “*Fitness is generally, in my opinion, a mobility thing”* (F398).

Meanwhile, unfit cows were those that had severe mastitis (“*If there is a coliform type of mastitis* [and] *I won’t be able to rehabilitate that animal for beef, then we make the decision to euthanase it*” [F833]), mobility issues (“*If she can’t walk* […] *she’s not suitable for the beef truck*,” [F663]), or other health conditions (“*If it’s an extreme case,* […] *if the animal has broken herself in any way we will euthanase for sure*” [F985]). Participants stated that unfit cows were either euthanased or treated until recovery. Some farmers also described using on-farm emergency slaughter for unfit cows (see Koralesky & Fraser 2018), but none had used it in recent years due to difficulties with organising the procedure. On-farm emergency slaughter in British Columbia is viewed by some in the dairy and veterinary community as a controversial practice, in part due to concerns that it may harm animal welfare by delaying the death of a suffering animal because it can take time to set up the on-farm emergency slaughter, which may contribute to its limited use (Koralesky & Fraser 2019). However, it can be a useful alternative slaughter method for farmers to manage cattle that are unfit for transport but fit for consumption because farmers will receive monetary compensation for the animal instead of needing to use euthanasia (McDermott *et al.* 2022). Koralesky and Fraser (2019) recommend the Government of British Columbia to provide further clarification on timing parameters and cow conditions that are allowable for on-farm emergency slaughter to encourage appropriate and timely use of on-farm emergency slaughter. Providing further clarity on on-farm emergency slaughter has the potential to encourage farmers to use the programme to manage unfit animals that fit the programme’s requirements and may thereby reduce the transport of unfit animals.

Similar to farmers, haulers determined fitness for transport by animal mobility and health. For example, hauler T023 described their decision-making process as follows:

*“The biggest thing is that if that animal’s got a bad limp, bad back,* [or] *weak back, I won’t put it on because they’re not going to make the trip. They’ll* […] *die on the truck. So, you’re looking for sick animals, you’re looking for animals that can’t stand very well.*”

The importance of mobility was also raised by Hauler T543:

“*It needs to be able to walk, like they need to be able to walk on. Like there’s some I’ve turned down and I said this cow’s three legged so I can’t transport it like that because by the time I get there, it’s going to be laying down.*”

In summary, both farmers and haulers typically defined unfit animals as those that have severe mobility or health issues. This may suggest that definitions of unfit animals within the dairying community may be fairly consistent, though this warrants further study. They also correspond to some of the definitions of unfit animals in the regulations, though they do not cover all conditions described in the new regulations. Rather, definitions of unfit cattle provided by both farmers and haulers reflect the requirements that were in place in the previous version of the Canadian transportation regulations which prohibited the transport of non-ambulatory animals. In the voice of one farmer “*It used to be not necessary for an animal to walk to* [go to] *a slaughter plant. It could be dragged onto a truck* […] *but now an animal needs to walk onto a trailer or transport vehicle to be deemed acceptable by the meat inspectors*” (F833). This focus on the previous, now outdated, definition was also reported by Hendricks *et al.* (2023) where participants discussed how management of cull cows has improved in recent years because the transport of non-ambulatory cows is no longer accepted. The seeming acceptance of the old regulations by participants in this study and the Hendricks *et al.* (2023) study implies that in time the new regulations may become more generally accepted, though this means that in the interim compromised cows will likely be subjected to non-compliant transport practices.

While shipment of compromised animals is allowed under the current regulations, special precautions must be taken. The participants in our study seemed unaware of which cattle would be considered compromised and did not report changing shipping or transport practices for such animals. Slight lameness that causes slightly imperfect locomotion, regardless of whether the cow feels pain or not, is considered a compromised condition under the regulations (HAR Part XII 136[1] 2022). However, some participants were unaware of this; for example, one farmer (F291) incorrectly defined a compromised cow and stated that shipping a chronically lame cow that was slightly lame was alright as long as she was not in pain:

*“Well, obviously* […] *you don’t want to and you never should be shipping a cow that’s compromised. Like if she’s sick or she’s or she’s limping.* […] *If you know she’s been a chronic lame cow, I mean, obviously there’s gonna be a little bit of lameness going on. But if she’s really super sore, no, then we’ll obviously try to deal with her, try to get her feeling better before* [transport].”

Cows in heavy lactation are also considered compromised (although the exact definition of heavy lactation is unclear in the regulations) and must be provided with adequate milking to prevent mammary engorgement throughout transport. Three of the six participating farmers dried off cows before transporting them, with two of them citing personal concerns about animal welfare and one citing the regulations. For example, farmer F663 said:

“[…] *If you’ve got a high-producing cow* […] *in the 45–50 litre plus range, we hate putting her on the truck because we don’t know when she’s going to be milked next.* […] *We do not normally send high-producing cows to the auction barn.*”

In addition to personal concerns about animal welfare and the regulations, some interviewed farmers and haulers described other motivators for providing for animal welfare. One such motivator was farmers’ pride in their animals. One farmer commented: “*I mean, I don’t want to take crap to the auction.* […] *There’s a sense of pride, you know what I mean? I don’t want to take an animal that I don’t think I see as fit”* (F985). Another farmer echoed these sentiments: “*We don’t want to be embarrassed about the cows we send away, is basically what it comes down to*” (F398).

Farmers also mentioned the importance of sending healthy animals for food safety and quality reasons: “*If that product is not fit for me to eat, then I don’t think I wouldn’t necessarily want to be serving it to anybody else*” (F985). Additionally, one farmer expressed the notion that shipping unfit animals could harm public perception of the industry: “*You can’t be sending lousy animals to* [slaughter]. *That shouldn’t happen. That is risk*[ing] *the whole industry by doing that*” (F663).

Meanwhile, three of the four interviewed haulers stated they felt a sense of responsibility over animal welfare as it related to trucking, and mentioned bedding, clean trailers, and climatic conditions. For example, hauler T023 expressed the connection between animal welfare and a clean trailer:

“*There should be a law that states that you have to have bedding at all times and cleaned out bedding for your next load* […] *I feel if I keep my trailer clean, animals like that.”*

Hauler T177 expressed the importance of hauling animals during cooler hours:

“*And I want to do the best I can.* […] *You know, if it’s going to be brutally hot, I mean, I’m not going to transport those animals, and* […] *I see even then if I can do it really late at night* […] *But yeah, I’m just not going to put them at risk.”*

One hauler (T023) linked animal welfare to their pride in their work and suggested that some haulers have poor practices because they do not care for animal welfare and do not have pride in their work:

*“I’m assuming just because they really don’t give a damn about livestock. That’s just my view on it. Honestly* […] *I still come from the old school driving where it was a respected thing.* […] *I believe a lot of drivers out there right now* […] *just see it as a job.* […] *They don’t love what they do. And where* [for] *myself, it was always something you did because you had passion in it, right?* […] *That is definitely an issue that the drivers aren’t caring for the animals that are on the back of them.”*

Similarly, Hendricks *et al.* (2023) also found that some haulers had negative opinions towards other haulers, felt that others operated dishonestly, were overly motivated by profit, and did not care for animals, and that such haulers should face consequences.

Safety is known to be a serious concern for livestock haulers, and this was echoed by some of the haulers in the current study. For example, hauler T147 stated:

“*The last thing that I want to happen is for there to be a cow that’s down in my trailer, and having issues that are dangerous to me, and dangerous to either that animal or other animals on the trailer.*”

Valadez-Noriega *et al.* (2018) reported that 28.4% of haulers in Mexico suffered accidents while handling livestock, and work as a hauler can involve long, irregular hours, with the potential for dangerous road accidents. Thus, shipping fit animals may promote the welfare of both haulers and the animals they transport, although further research is needed to confirm the connection between animal fitness for transport and worker safety.

Transporting cows that were fit for transport was also connected to a desire for business success. For example, one participant (T023) described how bringing mobile animals was important because it kept haulers from being blacklisted by buyers:

“*I hauled for about 4 years straight to* [a slaughter plant]. *They want them to be able to walk on and they walk off.* […] *I mean, you bring too many* [down cows] […], *you gotta be careful because they’ll ban you from coming in with another load. That’s not a good situation, right. You start losing money.*”

Providing for animal welfare during transport by keeping trailers clean was also connected to a desire for business success. Hauler T543 described how keeping a clean trailer was important for maintaining business with farmers: “*Yeah, if* [the] *trailer* [is] *really dirty like sloppy,* […] *the farmers don’t like it. But also in my experience, if I had a sloppy trailer, I would be worried that the cows would go down.*”

Providing the best care to animals is an expectation that the public has of farmers (see Cardoso *et al.* 2018). The public may hold haulers to the same expectations, given that haulers have a profound impact on the welfare of dairy cattle during transport while under their care. While farmers and haulers involved in our study described their practices to provide for animal welfare and were motivated by factors such as pride, responsibility, and safety, they still described a lack of compliance with the regulations. Indeed, one hauler participant (T249) stated that they had not changed any practices due to the regulations:

“*So do I know exactly what’s going on? I personally do not. My* [business partner] *may. He is the one who does the hauling mostly. The stuff* [in the regulations] *that is pertinent to what we do, yeah,* [it’s] *probably stuff we’re doing already. If it requires a whole bunch of extra paperwork, we’re probably not doing* [it].”

Social contracts are agreements between organisations and society that are intended to hold organisations accountable to what society expects of them (Gray *et al.* 1998). They typically relate to expectations of ethical conduct and may be reached through either implicit or explicit agreements between the parties included in the contract (Lacey & Lamont 2014). Industries that may harm the environment or animal welfare are often subject to a social contract, and research on such industries concludes that the public expects them to hold to a social contract or the industry risks losing its social licence to operate (Lacey & Lamont 2014; Hampton *et al.* 2020). Animal transport may be subject to a social contract, and the dairy industry may risk losing its social licence to operate if members of the public feel that farmers and haulers are not adequately providing for animal welfare.

### Cull dairy cow transport chain in British Columbia

The farmers and haulers involved in this study both cited challenges with the cull dairy cow transport chain in British Columbia that made compliance with the regulations difficult. These challenges included communication along the transport chain, limited local slaughter facilities, and inadequate transport infrastructure.

A lack of communication along the transport chain was identified as a major challenge, so individual actors along the chain often did not know exactly what happened next to the animal. In the words of farmer F398 when asked about the final destination of his cull cows: “*Some stay local. I know that. But I believe the majority go to* [a border state in the USA]*. But that’s just hearsay. I don’t know anything for a fact because once they’re dropped off at stockyards then they’re out of my hands, and I don’t know for a fact where they go.*”

Additionally, five of the six farmers interviewed in this study either did not give an estimate for how long cows were in transport because they did not know where cows ended up after being shipped or speculated that cows would be in transport for 24 h or less. Given that Stojkov *et al.* (2020) found that cull cows in British Columbia spend an average of 82 h in the transport and marketing system before slaughter, farmers may seriously underestimate how long their cows are in transport.

Similarly, haulers also appeared uncertain of what happens to a cow after delivery to the auction yard. Hauler T147 expressed:

“*You know, research does show sometimes they can’t get to slaughter within 36 hours. My own take* [is that auction yard staff] *are being really sensitive to the cows coming off* [Vancouver] *island and they’re assuring me that* […] *there’s a quick turnaround in the buying station to get them to slaughter, but that’s what I’m told. I can’t confirm that for sure.*”

Even if individuals may know about the next leg of the journey, they may have little control over it. Hauler (T543) discussed calf transport and how auction staff cannot control the loading and transport time of the animals:

“[The auction staff] *know that the calves go from* [British Columbia] *to Alberta. And they don’t stop. But he said it’s not really in his control. Like it’s hard for him to control what time they actually got on the truck.*”

Knowledge of a cull cow’s entire journey appears to be curtailed by a lack of communication between individuals along the transport chain, especially since cows may change ownership multiple times during transport, exacerbating communication issues (Stojkov *et al.* 2018). Without knowledge of the cow’s entire journey, individuals who are in temporary ownership (or custody) of the cow may not be able to make decisions that fully provide for the cow’s needs. For example, if a farmer shipped a lactating cow with the assumption that she will be slaughtered within 12 h, but the hauler was not aware that the cow was lactating, then the hauler may make decisions that result in the cow being in the system longer than 12 h and not being milked.

Increasing communication along the transport chain has the potential to change the behaviour of stakeholders along that chain. For example, if farmers’ shipping decisions are influenced by a misconception that transport times are short and cows will arrive at slaughter or auction in the same condition that they left the farm (Roche *et al.* 2020), then informing farmers about the transport chain may change shipping practices. In this study, farmer F833 described how knowing more about the cull cow transport chain led to a change in shipping practices:

“*I do not ship animals on Friday anymore because those animals hang around at the slaughter yard till* […] *Monday afternoon. So I only ship animals Monday to Thursday.* […] [I] *wasn’t aware of that until about a year ago, two years ago, that these animals that you ship on a Friday, they do not go to get slaughtered until the following Monday.*”

The new regulations require haulers to provide written information about animal condition at arrival, as well as the last time the animal was fed, watered, and rested, and the date and time of arrival to slaughter establishments or assembly centres (HAR Part XII 153[1], 2022). However, it is unclear if these requirements are being followed or whether they are being enforced by CFIA. Interviewed haulers only described filling out the Form 3, a mandatory livestock manifest in British Columbia that identifies the cow’s place of origin, gives a brief general description of the animal, and identifies the hauler (OII, n.d.); this form is then passed on to the next destination, whether an auction yard or slaughter plant (Livestock Identification Act 19 2007). Haulers may be unaware of the new requirements for record-keeping, suggesting further education about (and likely enforcement of) the regulations are needed to improve the management of animals during transport.

In the current study, most haulers expressed having positive, trusting, long-term relationships with the farmers they serve, and stated that they may discuss the cows’ condition before or at pick-up with the farmer, and accept or refuse animals accordingly. For example, hauler T147 described a time when a farmer asked for his opinion on a cow:

“*There was a time not long ago,* […] *and one of the guys said* […]*, “I’m just not sure about this cow.” And I said, “You know what, either make the decision not to send her or I’m going to come see her ahead of time.” And I did, went and saw her, and together, we decided that animal should* […] *be put down here* [and] *he had* [the] *opportunity to* […] *butcher her locally.*”

However, whilst some of the farmer participants reciprocated the notion that they had trusting, long-term relationships with their cattle haulers, they also stated that they felt confident about determining their cull cows’ fitness for transport and did not report discussing cow condition with their haulers. Dahl-Pederson *et al.* (2018a) found that Danish veterinarians, haulers, and farmers assessed dairy cattle fitness for transport differently, and a recent European Food Safety Authority (EFSA) scientific opinion piece also noted that a lack of clarity about the responsibilities of different actors in the transport chain (i.e. farmers, haulers) for determining fitness for transport further complicated decision-making about which cattle should be transported (EFSA Panel on Animal Health and Welfare 2022). It is possible that the cull dairy cow transport chain in British Columbia may also face the same issues, and encouraging discussions between farmers and haulers about cow fitness for transport may aid in aligning assessments of fitness for transport.

Some research suggests that haulers may feel pressured to take unfit animals to maintain good business relationships with farmers (Edwards-Callaway *et al.* 2019), which could limit open discussions about cow condition between farmers and haulers. For example, Hendricks *et al.* (2023) described how haulers in Atlantic Canada felt uncomfortable with having to refuse calves from farmers because they were too young (the new Canadian transport regulations prohibit transporting unweaned calves that are under eight days old). However, in the current study, we failed to hear any evidence of this as hauler T023 states:

“*I was expecting a lot more opposition from the farmer or even from the buyer when they buy them and actually went through the sale. But it actually caught on pretty quick. And people understood fairly quickly. They actually asked me, “Would you have a problem hauling that animal?” And that, once that happens,* […] *you’re not forced to. It’s up to you now, you know?”*

Perhaps due to regional differences, pressures to maintain business relationships may be less of an issue in British Columbia. However, equally likely is that despite our efforts we were not able to achieve data saturation given that we were only able to speak to four haulers.

In summary, communication along the cull dairy cow transport could be facilitated through means such as written records and discussions between responsible parties. As modern food supply chains are complex and dynamic, communication between all actors is needed to ensure that best practices are followed (Trienekens *et al.* 2012). These authors argue that transparency in agricultural supply chains is enabled by timely, accurate information exchange between supply chain actors, formal and informal governance mechanisms, and quality and safety standards for products. The cull dairy cow transport chain in British Columbia is lacking in information exchange capabilities, and this highlights the need for improved communication strategies to support animal welfare.

Aside from communication, both haulers and farmers discussed challenges with British Columbia’s slaughter and transport infrastructure that may also limit compliance with the regulations. One difficulty mentioned by some participants was a lack of local slaughter capacity and long waiting times. For instance, one farmer stated that “*You can wait a couple of months or six months or whatever* [for slaughter]*. And …*[finding] *a place to cut them up* […] *is equally difficult*” (F398).

Questions were also raised regarding why local direct-to-slaughter options are not more readily available:

*“What I would really like to see is if we had some direct-to-slaughter option, right?* […] *We’ve asked about it and* [the stockyard staff say]*, “Oh, just bring them to the stockyards.”* […] *But we’ve got a slaughterhouse here so it would be a lot better to just bring it to* [the slaughterhouse directly rather than] […] *bringing it* [to the stockyard and then back to the slaughterhouse]*”* (F398).

Improving local slaughter infrastructure and making alternative slaughter options, such as on-farm emergency slaughter, more available may encourage compliance with the regulations and give farmers more options when shipping cows.

Farmers and haulers also mentioned limited transport infrastructure that makes transport more difficult and may also lead to non-compliance with the regulations. Hauler T023 mentioned difficulties with limited rest stops and highway infrastructure:

“*I think the biggest issue is the fact that there’s not* […] *proper rest areas for the trucks to pull over* […] *I’ll pull over on the side of a highway* […] *because* [I’m] *out of hours. And then you’re expecting animals to be calm in a trailer when you’re right beside a highway with traffic going steady. Not to mention cold conditions, weather conditions and so forth on top of that.*”

Farmers were also concerned about rest stops and whether they existed, or whether current rest stop infrastructure would be adequate. For example, farmer F663 said:

“*If we’re going to be hauling all these animals from the Fraser Valley to Alberta,* […] *does something need to be set up halfway so after seven, eight hours these animals do get out of the truck and have access to exercise and food water and then get reloaded? Is that something that has to be built or someone has to build it* [and be] *in charge? But is that an option?”*

Inadequate slaughter and transport infrastructure in British Columbia was also reported in past studies (e.g. Stojkov *et al.* 2018) and clearly has yet to be remedied. There was some discussion as to whose responsibility it was to rectify these issues, but in all cases, interviewees felt solutions were the responsibility of someone else. For instance, one farmer suggested that challenges with slaughter infrastructure could be provided by entrepreneurs: “*I don’t know who would do it. But there’s always entrepreneurs out there that if they see an opportunity then they’ll take it*” (F398). Similarly, a farmer who mentioned challenges with inadequate rest stop infrastructure was unsure who could assume responsibility but was clear that it should be an external party: “*I’m sure* [someone’s] *thought of it, but it hasn’t been me. I’m not going to do it*” (F663). Given that these issues remain and remedying them may be necessary for compliance with the regulations, the dairy industry should consider playing a greater role in developing and implementing solutions.

### Limitations

These findings are not intended to be generalisable to the entire province of British Columbia, nor to the whole of Canada. However, given the similarities between the findings in this study and a study on cattle haulers in Atlantic Canada (Hendricks *et al.* 2023), it contributes to the growing body of evidence that challenges exist in cattle transport that are likely shared between different regions.

Animal transport is a sensitive topic, which likely explains why we experienced tremendous difficulties in the recruitment of haulers. Similar challenges were identified by Hendricks *et al.* (2023), who actively recruited for over six months and succeeded in recruiting only seven hauler participants. Lastly, this study consulted farmers and haulers, but the full journey of cull dairy cows also includes assembly points like auction markets and buying stations, as well as slaughterhouses. Further studies should include stakeholders at these stages for a more comprehensive understanding of the actors along the transport chain and their decision-making processes.

## Animal welfare implications and conclusion

This study aimed to capture the in-depth views of cattle haulers and dairy farmers to gain an understanding of their perspectives on cull dairy cow transport, as well as the impact of new federal regulations on animal transport. Amongst those interviewed for this study, barriers to compliance with the regulations appear to be little knowledge of the regulations, mixed or negative attitudes towards the regulations, a lack of communication along the transport chain, and slaughter and transport infrastructure. If these findings are confirmed in larger studies, it may suggest a need for increased education and engagement with farmers and haulers about the regulations, stronger enforcement of regulatory requirements for written communication in the transport chain, and improved slaughter and transport infrastructure. Although the regulatory update may be a positive step towards improving the welfare of cull dairy cattle during transport, this study provides a first glance into potential limitations of the regulations and barriers to compliance for cattle haulers and dairy farmers.
